# Antitumor Activity of Ligustilide Against Ehrlich Solid Carcinoma in Rats via Inhibition of Proliferation and Activation of Autophagy

**DOI:** 10.7759/cureus.40499

**Published:** 2023-06-16

**Authors:** Afnan F Alshehri, Ahmed E Khodier, Mohammed M Al-Gayyar

**Affiliations:** 1 PharmD, University of Tabuk Faculty of Pharmacy, Tabuk, SAU; 2 Pharmacology, Horus University, Faculty of Pharmacy, New Damietta, EGY; 3 Biochemistry, Mansoura University Faculty of Pharmacy, Mansoura, EGY; 4 Pharmaceutical Chemistry, University of Tabuk Faculty of Pharmacy, Tabuk, SAU

**Keywords:** mammalian target of rapamycin (mtor), ki67, b-cell lymphoma 2 (bcl2), beclin 1, 5' adenosine monophosphate-activated protein kinase (ampk)

## Abstract

Background

Cancer is the second-leading cause of death worldwide. According to a 2018 WHO report, 9.6 million deaths occurred globally due to cancer. Ehrlich carcinoma is characterized by rapid proliferation and a short survival time. Ligustilide is a phthalide derivative and is one of the main compounds in Danggui essential oil and *Rhizoma Chuanxiong*. It has many protective effects, such as anticancer, anti-inflammatory, antioxidant, and neuroprotective effects.

Aims

We conducted this study to investigate the antitumor activity of ligustilide against Ehrlich solid carcinoma (ESC) in rats by affecting beclin 1, mammalian target of rapamycin (mTOR), B-cell lymphoma 2 (BCL2), and 5' AMP-activated protein kinase (AMPK).

Materials and methods

Twenty rats were intramuscularly implanted in the thigh of the left hind limb with a 200-µL tumor cell suspension in PBS containing 2 × 10^6^ cells. After eight days of inoculation, 10 rats out of the 20 were treated with oral 20 mg/kg ligustilide daily. At the end of the experiment, samples of muscles with ESC were separated. Sections prepared from the muscle samples with ESC were immunohistochemically stained with anti-Ki67 antibodies. Another part of the muscle samples with ESC was used to assess gene expression and protein levels of beclin 1, mTOR, BCL2, and AMPK.

Results

Treatment of carcinoma rats with ligustilide elevated the mean survival time and reduced tumor volume and weight. Moreover, examination of tumor tissue stained with hematoxylin/eosin showed an infiltrative, highly cell-dense mass supported by a small to moderate amount of fibrovascular stroma and intersected with multifocal myofibril necrosis. Treatment with ligustilide ameliorated all these effects in the carcinoma group without affecting the control group. Finally, treatment with ligustilide significantly decreased the expression of beclin 1, mTOR, and AMPK associated with elevated expression of BCL2.

Conclusions

Our study aimed to explore the potential chemotherapeutic activity of ligustilide against ESC. We found that ligustilide effectively reduced tumor size and weight, indicating its antineoplastic activity against ESC. We further investigated that ligustilide inhibits cell proliferation by suppressing Ki67 and mTOR and activates autophagy through beclin 1 activation. Moreover, ligustilide inhibits apoptosis by upregulating BCL2. Finally, ligustilide reduced the expression of AMPK, preventing its ability to promote tumor cell growth.

## Introduction

Cancer is a severe disease and the second-leading cause of death worldwide. According to a WHO report in 2018 [1], it is complicated to treat due to many characteristics related to the pathology of the disease and the side effects of the chemotherapy treatment, including loss of hair, fatigue, nausea, and many others [2]. Ehrlich carcinoma is a spontaneous murine mammary adenocarcinoma. Characterized by rapid proliferation and a short survival time, it is usually utilized in many tumor and chemotherapy studies [3]. They are maintained by intraperitoneal passages in an ascitic form (EAC). Ehrlich tumor cells can also develop into solid tumors (ESC) when injected subcutaneously [4].

Beclin 1 is a member of class 3 phosphatidylinositol-3-kinase (PI3KC3). It is a tumor suppressor gene and a primary regulator of autophagy. High values of beclin 1 are associated with a good prognosis in patients with colorectal cancer [5]. It reacts with B-cell lymphoma-2 (BCL-2) to enhance the process of apoptosis through the expression of caspases [6]. In parallel, the mammalian target of rapamycin (mTOR) is an autophagy inhibitor through the enhancement of the signaling pathways of mitogen-activated protein kinase (MAPK) and protein kinase B (AKT) [5].

Ligustilide is a phthalide derivative. It is a leading and most effective compound of Danggui essential oil and Chuanxiong Rhizoma, traditional Chinese herbals. Ligustilide could be a promising compound due to its pharmacological properties; it has antitumor, anti-inflammation, antioxidant neuroprotection, and vasodilatation activities [7]. Ligustilide was also reported to treat hepatocellular carcinoma by regulating cancer cells and the crosstalk between tumor cells and macrophages in the tumor microenvironment [8]. Our objective was to investigate the potential chemotherapeutic impact of ligustilide on EAC in rats by targeting beclin 1, mTOR, B-cell lymphoma 2 (BCL2), and 5' AMP-activated protein kinase (AMPK).

This article was previously presented as a meeting abstract at the 2023 Dubai International Pharmaceuticals and Technologies Conference and Exhibition (DUPHAT) on January 10-12, 2023.

## Materials and methods

Animals and treatment protocol

We conducted an experiment on 30 Sprague-Dawley rats weighing 180-200 g, maintaining them under standard temperature conditions with a regular 12-hour light/12-hour dark cycle. The Research Ethics Committee of Horus University, Faculty of Pharmacy, approved our working protocol with the number P2023-003. We divided the rats into three groups, each consisting of ten rats.

The Control Group

The rats in this group were untreated during the entire experiment period.

ESC Group

Rats in the ESC group received an intramuscular injection of 0.15 ml of 2 × 106 Ehrlich cells in the thigh of the left hind leg and left for four weeks without any treatment [9].

ESC Treated With Ligustilide Group

After induced ESC in rats, once a solid tumor appeared on day 8, rats were administered 20 mg/kg ligustilide (Sigma Aldrich Chemicals Co., Burlington, MA, USA) by oral gavage and indicated as day 0. The rats were then treated with ligustilide for three more weeks.

Sample collection 

The tumor area on the left hind leg's thigh was removed, measured, and weighed. For morphological and immunohistochemistry studies, a portion of the muscle tissue with ESC was preserved in 10% buffered formalin. Another portion was homogenized in an ice-cold sodium-potassium phosphate buffer and then stored at −80 °C.

Immunohistochemistry 

Our group previously described the process of performing immunohistochemistry [10-12]. To begin, we cut 5-µm-thick paraffin sections from a block of muscle tissues. Next, we immunostained the cells with monoclonal anti-Ki67 from Santa Cruz Biotechnology, Inc., Dallas, TX, USA, at 4 °C. Afterward, we incubated the sections with a horseradish peroxidase conjugate antibody and counterstained them with hematoxylin. Finally, the cells were examined in a masked manner.

Biochemical investigations using enzyme-linked immunosorbent (ELISA)

The assessment of AMPK (Catalog number MBS1602983), beclin 1 (Catalog number MBS2706719), Ki67 (Catalog number MBS705024), BCL2 (Catalog number MBS2515143), and mTOR (Catalog number MBS744326) was conducted using commercially available ELISA kits from MyBioSource, Inc., San Diego, CA, USA, in accordance with the manufacturer's instructions.

RT-PCR investigations

To analyze gene expression in rat muscle, we followed our group's established protocol [13-16]. We measured the levels of AMPK, beclin 1, BCL2, and mTOR mRNA using specific PCR primers listed in Table 1. We used GAPDH as a housekeeping gene and internal reference control.

**Table 1:** Primer sets used to detect gene expression in rats.

Gene symbol	Primer sequence from 5′- 3′	Accession number
GAPDH	F: 5`-CCATCAACGACCCCTTCATT-3` R: 5`-CACGACATACTCAGCACCAGC-3`	NM_017008.4
AMPK	R: 5`CCTTCGGCAAAGTGAAGATTGG-3` R: 5`ATGAAGGAACCCGTTGGAGG-3`	NM_023991.1
BCL2	F: 5`-AGTTCGGTGGGGTCATGTGTG-3` R: 5`-CCAGGTATGCACCCAGAGTG-3`	NM_016993.2
mTOR	F: 5′-CTGCACTTGTTGTTGCCTCC-3′ R: 5′-ATCTCCCTGGCTGCTCCTTA-3′	NM_019906.2
Beclin 1	F: 5`-CTCGTCAAGGCGTCACTTCT-3` R: 5`-CCTCCATTCTTTAGGCCCCG-3`	NM_053739.2
Ki67	F: 5`-TTCCAGACACCAGACCATGC-3` F: 5`-GGGTTCTAACTGGTCTTCCTGG-3`	NM_001271366.1

Statistical analysis

In presenting quantitative variables, we utilized the mean ± standard error. To compare groups, we utilized a one-way analysis of variance (ANOVA) followed by a post hoc Bonferroni correction test. All statistical analyses were performed using SPSS version 20 (Chicago, IL, USA). We defined statistical significance as P < 0.05.

## Results

Antitumor activity of ligustilide

**Figure 1:**
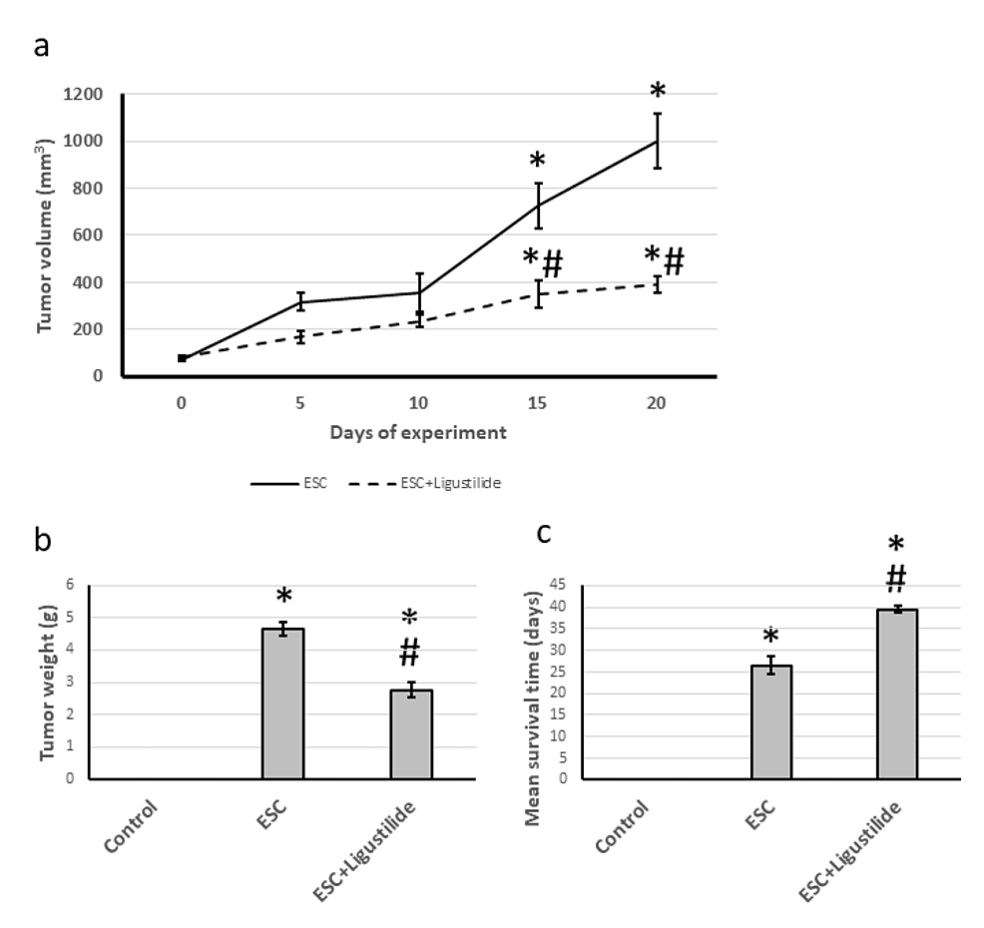
The influence of ESC induced in rats and treatment with 20 mg/kg ligustilide on tumor volume (a), tumor weight (b), and mean survival time (c). *Represented a significant difference when compared to the control group at a significance level of p<0.05. Similarly, ^#^represented a significant difference when compared to the ESC group at a significance level of p<0.05. ESC: Ehrlich solid carcinoma.

First, we examined the direct effect of ligustilide on the tumor. We found that ligustilide significantly reduced the tumor weight and size by about 60%. In addition, ligustilide significantly increased the survival of rats from 26 days to 41 days (Figure 1).

Effect of ligustilide on tumor-induced elevation in cell proliferation 

**Figure 2:**
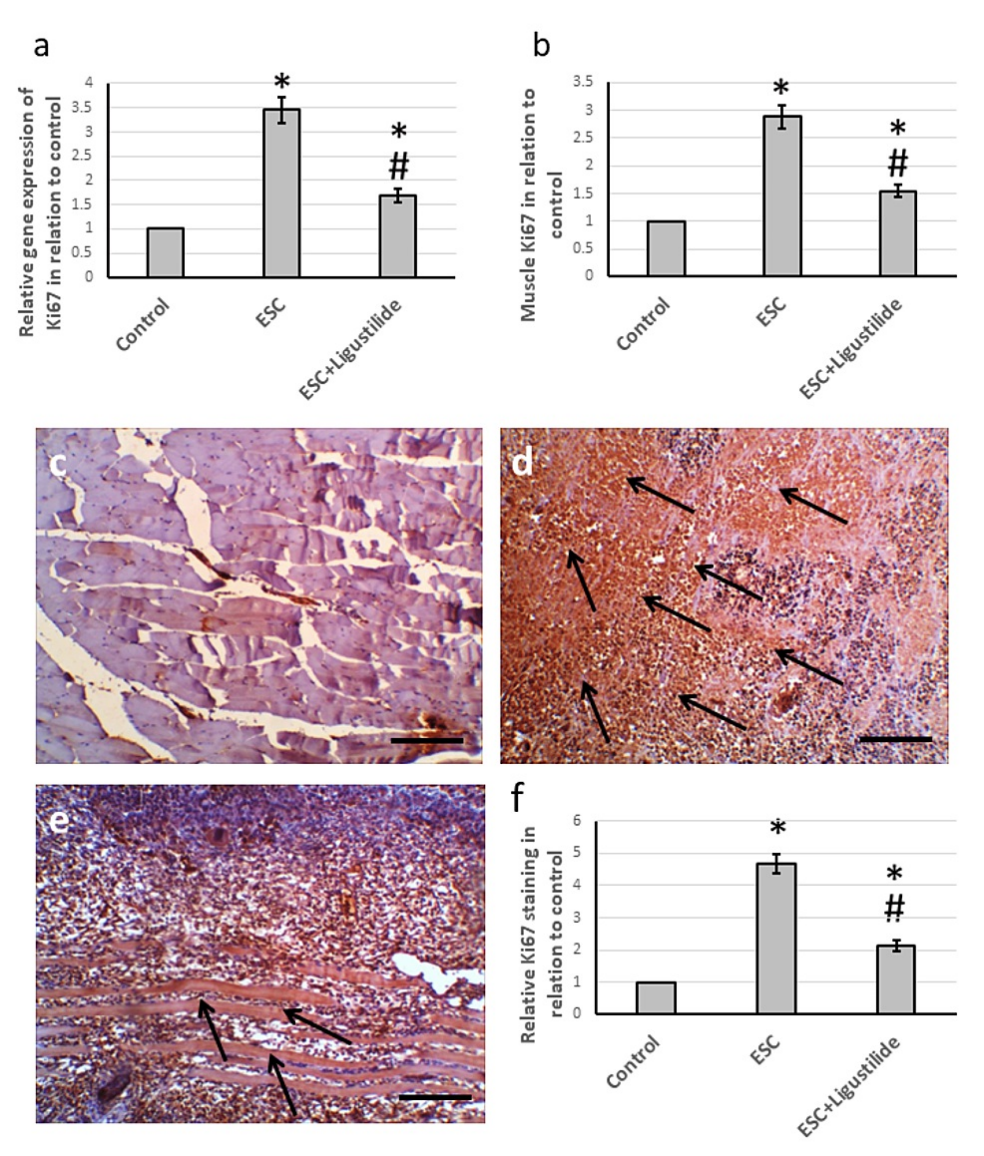
The influence of ESC induced in rats and treatment with 20 mg/kg ligustilide on the Ki67 gene expression (a) and protein levels (b). Muscle sections stained with anti-Ki67 antibodies in the control group (c), ESC group (d), and ESC group treated with ligustilide (e). Immunohistochemistry score of positive staining (f). The immunohistochemistry score was calculated by dividing the total area stained with anti-Ki67 by the total tissue area in the field, measured in 10 different fields of each animal section. *Represented a significant difference when compared to the control group at a significance level of p<0.05. Similarly, ^#^represented a significant difference when compared to the ESC group at a significance level of p<0.05. Scale bar 100 μm. ESC: Ehrlich solid carcinoma.

Ki67 is a proliferation marker strongly associated with tumor cell proliferation [17]. ESC resulted in an elevation in the gene expression of Ki67 and its muscle protein level as compared with the control group. In addition, examination of muscle sections immuno-stained with anti-Ki67 revealed an increase in immuno-staining in muscle sections from ESC, which was reduced in sections from rats treated with ligustilide. In addition, ligustilide decreased the analysis of the anti-Ki67 stained area (Figure 2).

Effect of ligustilide on beclin 1

**Figure 3:**
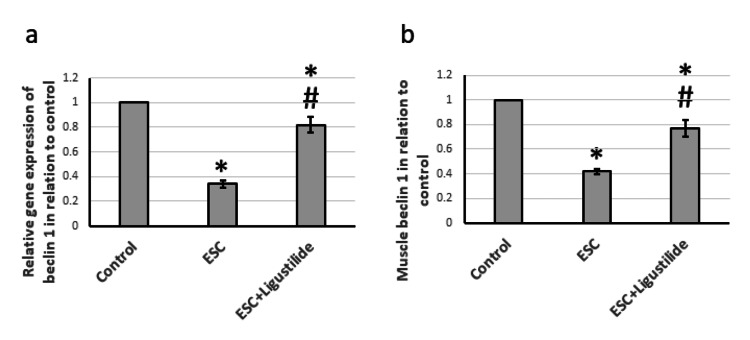
The influence of ESC induced in rats and treatment with 20 mg/kg ligustilide on beclin 1 gene expression (a) and protein levels (b). *Represented a significant difference when compared to the control group at a significance level of p<0.05. Similarly, ^#^represented a significant difference when compared to the ESC group at a significance level of p<0.05. ESC: Ehrlich solid carcinoma.

After finishing the examination of the antitumor activity of ligustilide, we looked inside the molecular mechanism of action to investigate the pathological effects on autophagy, a complex catabolic program for lysosomal degradation of proteins and other subcellular constituents that is activated in response to nutrient deprivation, leading to the recycling of organelles and other cytoplasmic substances to provide metabolic precursors. Beclin 1 is the first autophagy core-machinery protein identified as a caspase substrate [18]. ESC reduced gene expression and muscle protein levels of beclin 1 compared to the control rats. Treatment of ESC rats with ligustilide significantly increased the expression of beclin 1, but still less than the control level. Therefore, ESC reduced autophagy, which was restored by treatment with ligustilide (Figure 3).

Effect of ligustilide on BCL2

**Figure 4:**
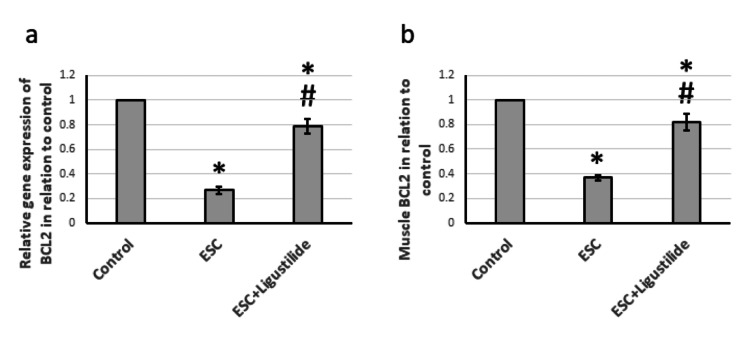
The influence of ESC induced in rats and treatment with 20 mg/kg ligustilide on BCL2 gene expression (a), and protein levels (b). *Represented a significant difference when compared to the control group at a significance level of p<0.05. Similarly, ^#^represented a significant difference when compared to the ESC group at a significance level of p<0.05. ESC: Ehrlich solid carcinoma, BCL2: B-cell lymphoma 2.

BCL2 is the founding member of the BCL family of regulator proteins that produce anti-apoptotic effects [19]. ESC resulted in reduced gene expression and protein levels of BLC2 compared to the control rats. Treatment with ligustilide significantly increased the expression of BCL2, but still less than the control level (Figure 4). Therefore, ESC reduced cell protection against apoptosis, which was restored by treatment with ligustilide.

Effect of ligustilide on mTOR

**Figure 5:**
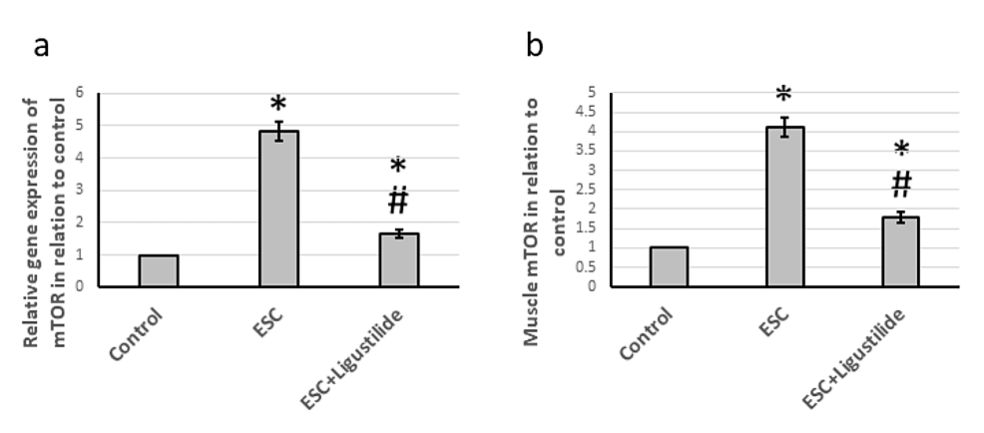
The influence of ESC induced in rats and treatment with 20 mg/kg ligustilide on mTOR gene expression (a), and protein levels (b). *Represented a significant difference when compared to the control group at a significance level of p<0.05. Similarly, ^#^represented a significant difference when compared to the ESC group at a significance level of p<0.05. ESC: Ehrlich solid carcinoma, mTOR: mammalian target of rapamycin.

mTOR functions as a serine/threonine protein kinase that regulates cell growth, cell proliferation, cell motility, cell survival, protein synthesis, autophagy, and transcription [20]. In ESC, mTOR had elevated gene expression and protein levels compared to the control group. Treatment with ligustilide significantly reduced the expression of mTOR but was still higher than the control level (Figure 5).

Effect of ligustilide on AMPK

**Figure 6:**
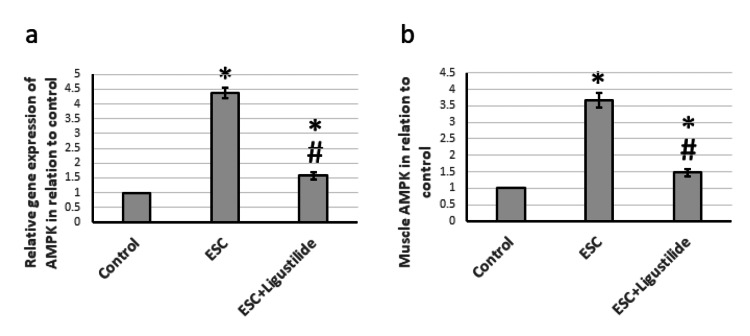
The influence of ESC induced in rats and treatment with 20 mg/kg ligustilide on AMPK gene expression (a) and protein levels (b). *Represented a significant difference when compared to the control group at a significance level of p<0.05. Similarly, ^#^represented a significant difference when compared to the ESC group at a significance level of p<0.05. ESC: Ehrlich solid carcinoma, AMPK: 5'-adenosine monophosphate-activated protein kinase.

Next, we investigated the effect on AMPK, a highly conserved sensor of low intracellular ATP levels that is rapidly activated after nearly all mitochondrial stresses, even those that do not disrupt the mitochondrial membrane potential [21]. Figure 6 revealed that ESC resulted in elevated gene expression and protein levels of AMPK. Treatment with ligustilide significantly reduced the expression of AMPK, but it was still higher than the control level. Therefore, ESC activated tissue stress, which was reversed by treatment with ligustilide.

## Discussion

According to a 2018 report by the World Health Organization, cancer is the second leading cause of death globally, responsible for over 9.6 million deaths. It is projected to increase to 21.4 million deaths worldwide by 2030 [22]. Cancer treatment often results in severe symptoms, emotional distress, and physical limitations, which can lead to a decreased quality of life and depression. Additionally, chemotherapy can be extremely expensive and cause serious side effects. The aim of our current research is to investigate the potential chemotherapeutic effects of ligustilide. For our study, we utilized the ESC tumor model, which is characterized by rapid growth [23]. We implanted Ehrlich cancer cells subcutaneously in the thigh of the left hind leg, resulting in the appearance of a lump that increased in size over several weeks due to tumor growth. The diagnosis was confirmed through the separation of the tumor after animal sacrifice and inspection of micro-images stained with anti-Ki67 antibodies.

In an effort to find a chemotherapeutic agent, the effectiveness of ligustilide was investigated. Previous studies had shown that ligustilide had anti-inflammation, antioxidant, and antitumor properties [7]. When tested on ESC rats, ligustilide significantly reduced tumor volume and weight while increasing the mean survival time from 26 to 41 days. Additionally, examining the micro-images of the rats treated with ligustilide revealed a decrease in immunostaining with Ki67 antibodies. Previous studies had shown that ligustilide had chemotherapeutic properties against bladder cancer [24], oral cancer cells [25], breast cancer [26], and prostate cancer [27] due to its antioxidant and antiapoptotic properties. However, this is the first study to show that ligustilide is effective in reducing ESC.

We examined muscle sections stained with Ki67, which is a proliferative marker. The Ki67 protein has long been used as a prognostic marker for cancer treatment [17]. It has also been studied in many experimental models of cancer [28,29]. Ki67 is overexpressed throughout all the active phases of the cell cycle. It was previously commonly used as a marker of Ehrlich tumor proliferation. We discovered elevated gene expression and protein levels of Ki67 associated with increased immune staining of Ki67 in ESC rats. Treating rats with ligustilide significantly reduced the expression of Ki67. Ligustilide was previously reported to alleviate insulin resistance and lipid accumulation in experimentally induced diabetes mellitus in rats through activation of AMPK [30]. However, this is the first research to report the ability of ligustilide to reduce the expression of Ki67 in ESC.

Beclin-1 is a critical protein that controls the activity of lipid kinase, leading to the activation of autophagy. It interacts with the sodium-potassium ATPase pump, regulating the survival and death cycles of cells [31]. Our data suggest that ligustilide enhances autophagy and attenuates tissue stress. Activation of beclin 1 can inhibit tumorigenesis by eliminating damaged organelles and other cellular components. In addition, beclin 1 is included in the formation of class III PI3K complexes, which are essential for autophagy initiation [32]. Ligustilide was previously reported to activate autophagy in A7r5 fibroblast-like cell cells [33], PC12 brain cells [34], and breast cancer cells [26].

We investigated the apoptotic pathway as a possible mechanism for the pathogenicity of ESC. This process is regulated by a balance of proapoptotic proteins (BAX, BAK, and p38) and antiapoptotic proteins (BCL2, BCLX, and MCl-1) within cells. Our findings revealed an overexpression of BAX and a downregulation of BCL2, causing an imbalance that can affect apoptosis and trigger caspase activation. BCL2 is a well-known anti-apoptotic compound that protects cells from apoptosis by regulating antioxidant activities. On the other hand, BAX promotes apoptosis by activating cytochrome C and caspase-3 [19]. However, treatment with ligustilide in rats effectively upregulated BCL2.

Our investigation examined the impact of ligustilide on cell function by analyzing the role of mTOR, which is a serine/threonine kinase. It is a key factor in autophagy induction and plays a vital role in cell growth, proliferation, autophagy, and survival. Promising results have been seen from testing mTOR inhibitors against tumor cells [20]. We found that ligustilide significantly reduced the ESC-induced elevation in the expression of mTOR. Ligustilide was reported previously to inhibit mTOR signaling in angiotensin II-induced A7r5 cell autophagy [33] and protect PC12 cells from oxygen-glucose deprivation/reoxygenation-induced apoptosis [34]. However, no previous report illustrated the ability of ligustilide to inhibit mTOR in ESC.

AMPK serves as a crucial regulator of energy metabolism, controlling anabolic pathways like fatty acid synthesis and promoting catabolic pathways like fatty acid oxidation to maintain ATP levels in cells [21]. While some studies suggest that it can hinder cancer cell proliferation, recent research indicates that it can also enhance tumor cell growth by maintaining cell energy in certain models. The hyperactive signaling of AMPK plays a dominant role in the biology of cancer and its progression. The roles of AMPK and the mTOR complex are emerging in the molecular pathways that connect autophagy and senescence [35]. Our findings show that ligustilide significantly reduces the expression of AMPK induced by ESC. Ligustilide was previously reported to decrease the expression of AMPK in ischemic stroke [36], cardiomyocyte dysfunction [37], and memory deficit [38]. However, no previous report has demonstrated the ability of ligustilide to inhibit AMPK in ESC.

**Figure 7:**
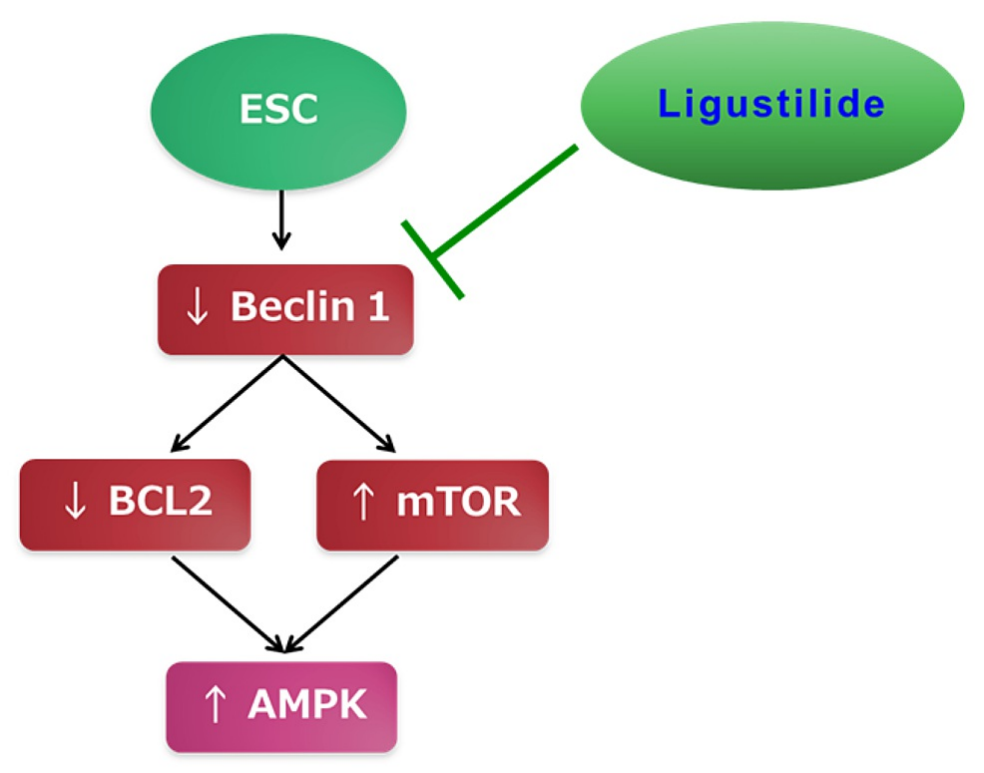
The mechanism of the protective effects of ligustilide in ESC. ESC: Ehrlich solid carcinoma, AMPK: 5'-adenosine monophosphate-activated protein kinase, BCL2: B-cell lymphoma 2. Image Credits: The authors of the manuscript.

Ligustilide is a promising option for chemotherapy due to its natural occurrence, affordability, and safety. Figure 7 summarizes the mechanism of its chemotherapeutic effects in ESC. However, the current research has certain limitations. For instance, rats have different metabolic pathways than humans, which can lead to varying effects of certain drugs. Additionally, there are multiple animal models for inducing cancer, but we only used one method in our study.

## Conclusions

Our study aimed to explore the potential chemotherapeutic activity of ligustilide against ESC. We found that ligustilide effectively reduced tumor size and weight, indicating its antineoplastic activity against ESC. Upon further investigation, we discovered that ligustilide inhibits cell proliferation by suppressing Ki67 and mTOR and activates autophagy through beclin 1 activation. Moreover, ligustilide inhibits apoptosis by upregulating BCL2. Finally, ligustilide reduced the expression of AMPK, preventing its ability to promote tumor cell growth. AMPK plays a controversial role in tumorigenesis.
